# Microcephaly, Agenesis of the Corpus Callosum, and Suspected Blake Pouch Cyst Presenting With Failure to Thrive in an Infant

**DOI:** 10.7759/cureus.112585

**Published:** 2026-07-13

**Authors:** Holly Ingram, Kelson Knighton, Nicole Skalka, Daniel Chappell

**Affiliations:** 1 Neurology, Rocky Vista University College of Osteopathic Medicine, Ivins, USA; 2 Otolaryngology - Head and Neck Surgery, Rocky Vista University College of Osteopathic Medicine, Ivins, USA; 3 Dermatology, Rocky Vista University College of Osteopathic Medicine, Ivins, USA; 4 Family Medicine, Tanner Clinic, Farmington, USA

**Keywords:** agenesis of corpus callosum, craniosynostosis, developmental delay, failure to thrive, infant neurodevelopment, microcephaly

## Abstract

Infants presenting with microcephaly may present with misshapen head abnormalities that warrant surgical intervention or further investigation. Cranial growth restriction may occur secondary to impaired cerebral development with patent sutures, a diagnostic distinction with critical implications for management. Craniosynostosis, characterized by premature fusion of cranial sutures, is a common consideration in infants presenting with abnormal skull morphology and microcephaly. Agenesis of the corpus callosum (ACC) is a congenital brain malformation that may present with microcephaly, failure to thrive, and early developmental delay, potentially mimicking craniosynostosis on physical examination.

A two-month-old male infant born at term via uncomplicated vaginal delivery presented with microcephaly, failure to thrive, and unmet developmental milestones. Based on the infant's abnormal head shape, he was suspected of having craniosynostosis. Growth parameters demonstrated severe restriction: length 2nd percentile, weight less than the 1st percentile, and head circumference less than the 1st percentile, with minimal interval growth from birth to 2 months. Physical examination revealed palpable ridging along the coronal sutures and a small, flat anterior fontanelle. Neurologic assessment demonstrated absent social smile and poor eye contact despite intact pupillary responses, full conjugate eye movements, and normal extremity movement. Feeding difficulties requiring post-prandial suctioning raised concern for laryngomalacia or another upper airway obstruction with a possible need for nasogastric tube placement. Computed tomography demonstrated no evidence of craniosynostosis. Subsequent magnetic resonance imaging revealed complex ACC with colpocephaly, parallel lateral ventricles, and a Blake pouch cyst (BPC). Pediatric neurosurgical evaluation concluded that cranial growth restriction was secondary to impaired cerebral development rather than primary suture pathology. Genetic evaluation and neurodevelopmental follow-up were initiated.

This case illustrates a critical diagnostic pitfall: cranial suture ridging and microcephaly do not always indicate craniosynostosis. In infants with failure to thrive, abnormal head growth and underlying brain malformations, such as ACC, may produce secondary cranial restriction with patent sutures. The presence of congenital microcephaly and early developmental concerns, such as absent social interaction, should heighten suspicion for underlying structural brain abnormalities. Early recognition of this distinction is essential to initiate appropriate genetic evaluation and neurodevelopmental monitoring. Complex ACC with associated anomalies warrants a comprehensive workup with multidisciplinary evaluations, as outcomes depend significantly on the presence of additional malformations and underlying genetic etiology.

## Introduction

Craniosynostosis is a condition characterized by premature fusion of one or more cranial sutures, resulting in abnormal skull growth and potential neurologic complications. The condition occurs in approximately 5.9 per 10,000 live births worldwide and may occur in isolated or syndromic forms [1]. Early diagnosis is important because untreated craniosynostosis may lead to elevated intracranial pressure and developmental impairment. However, abnormalities in cranial shape or size do not always represent true suture fusion. Cranial growth is closely linked to cerebral expansion, and impaired brain development can result in secondary cranial restriction even when sutures remain patent. Agenesis of the corpus callosum (ACC) is a congenital brain malformation resulting from the failure of commissural fibers to cross the midline during early fetal development. Formation is generally complete between 18 and 20 weeks of gestation [2]. ACC is a common congenital brain malformation, with an estimated incidence of approximately 1 in 4,000 individuals in the general population [2]. The clinical presentation of ACC varies widely and may include developmental delay, seizures, feeding difficulties, and structural craniofacial abnormalities [3]. 

Recent longitudinal research has demonstrated that infants with ACC may exhibit developmental differences as early as 6 months of age, with communication skills affected earliest, followed by motor and daily living skills by 12 and 18 months [4]. Importantly, children with ACC have also been shown to have increased likelihood for autistic behaviors, making early recognition of social engagement deficits clinically significant [4]. Information on the neurodevelopment of patients younger than six months is lacking. 

Blake pouch cyst (BPC) is a posterior fossa malformation within the Dandy-Walker continuum, resulting from a failure of fenestration of the posterior membranous area during embryogenesis [5]. BPC may occur in isolation or in association with other central nervous system anomalies. When present with ACC, the combination suggests a broader disruption of midline brain development during the first trimester [6].

We present a case of an infant initially evaluated for microcephaly and failure to thrive with a suspected craniosynostosis who was subsequently found to have complex ACC with colpocephaly, suggesting a BPC, illustrating the diagnostic challenges in distinguishing primary cranial suture pathology from secondary cranial growth restriction. This case also highlights the importance of recognizing early developmental warning signs in infants with congenital brain malformations.

## Case presentation

Patient information 

A two-month-five-day-old male infant presented for evaluation of failure to thrive and concerns for abnormal cranial growth. The patient was born near term via an uncomplicated vaginal delivery. He is the second child born to his parents; his older brother is healthy with no known developmental or neurologic conditions. There was no reported family history of congenital brain malformations, genetic syndromes, or craniosynostosis. Per the patient’s mother, prenatal history was unremarkable, with no reported maternal infections, teratogen exposures, or known abnormalities detected on prenatal ultrasound.

Clinical findings

The patient's parents reported persistent feeding difficulties characterized by significant congestion during feeding and the need for suctioning after nearly every meal. Symptoms raised suspicion for possible laryngomalacia or other upper airway obstruction. They had worked with a lactation consultants to help with possible causes of feeding difficulties. The family also expressed concerns about the patient’s limited social engagement. When asked about his development of a “social smile” or his ability to smile back in response to parents' smiles or voice, they deny seeing any engagement.

Growth measurements demonstrated significant growth restriction. At birth, he weighed 7 lbs 8 oz, and his head circumference measured approximately 13 inches, already below the 5th percentile, indicating congenital microcephaly. At the 1-month appointment, the patient measured 20.75 inches in length (7th percentile), weighed 8 lb 3 oz (4th percentile), and had a head circumference of 13.5 inches (less than 1st percentile). At the current visit, the patient was 2 months 5 days of age and measured 21.5 inches in length (2nd percentile), weighed 8 lb 3 oz (less than 1st percentile), and had a head circumference of 13.78 inches (less than 1st percentile).

**Figure 1:**
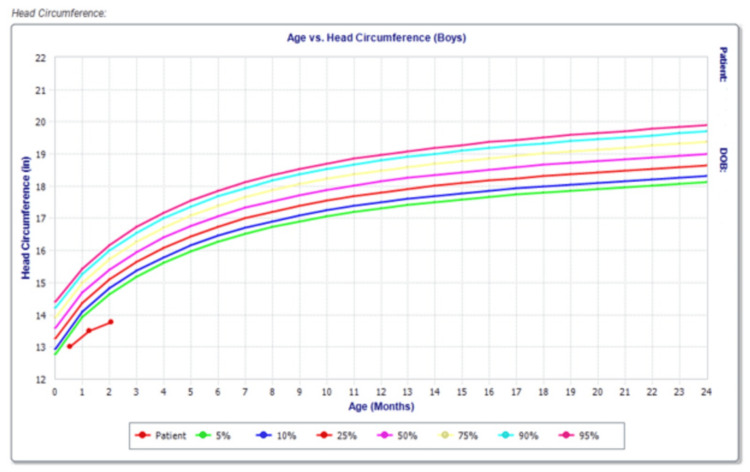
Head circumference growth chart (boys) demonstrating persistent severe microcephaly The patient’s measurements (red line) at birth, 1 month, and 2 months of age consistently fall below the 5th percentile, with minimal interval growth. Standard percentile curves are shown for reference.

Comparison with measurements from the previous visit showed persistent microcephaly and no weight gain over one month. Serial measurements demonstrated minimal interval head growth with a widening gap from normal growth curves (Table 1, Figure 1).

**Table 1:** Serial growth measurements HC = head circumference; %ile = percentile; mo = month; d = days

Age	Length (in)	Length %ile	Weight (lbs)	Weight %ile	Head Circumference (in)	HC %ile
Birth	-	-	7 lb 8 oz	-	13.0	<5th
1 month, 8 days	20.75	7th	8 lb 3 oz	4th	13.5	<1st
2 months, 5 days	21.5	2nd	8 lb 15 oz	<1st	13.78	<1st

**Figure 2:**
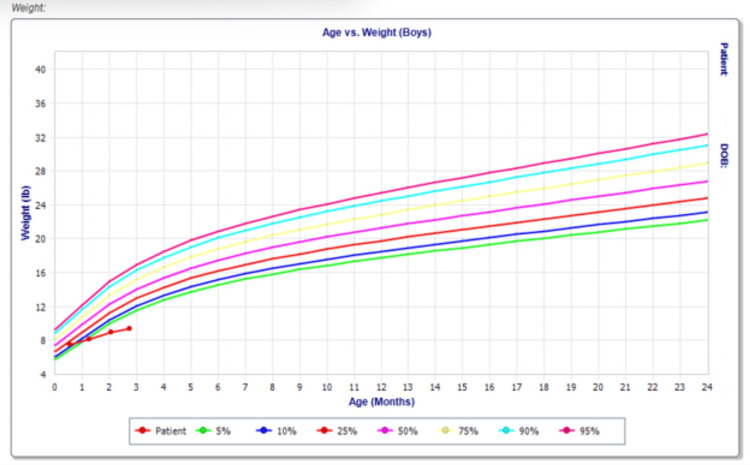
Weight growth chart demonstrating the infant's weight trajectory over time Weight has remained below the 5th percentile with a flat growth curve, meeting criteria for failure to thrive. Standard percentile curves are shown for reference.

At follow-up at approximately 3 months of age, the infant demonstrated persistent failure to thrive with weight trending flat and remaining below the 5th percentile (Figure 2). Feeding difficulties continued with persistent feed refusal and distress after feeding, particularly with increased volume; the infant tolerated only 1.5-2.5 ounces per feed. Multiple interventions were attempted, including lactation support, pacing, and timing adjustments, without improvement. Given the lack of response to conservative measures, nasogastric tube placement was being considered for nutritional support. The infant has continued follow-up with pediatric neurology and is scheduled for genetics, ophthalmology, and further gastroenterology evaluation.

Physical examination revealed palpable ridging along the coronal sutures and a small anterior fontanelle with flattening of the cranial vault, raising concern for possible craniosynostosis.

Neurologic examination demonstrated pupils that were equal and reactive, with full and conjugate movements. The patient was able to move all extremities symmetrically with age-appropriate tone and reflexes. However, developmental assessment revealed concerning findings: the infant did not track faces consistently and did not demonstrate reciprocal social engagement during the examination. The mother expressed concern that, in comparison to her older son at this age, her infant has never smiled and continues to have poor eye contact. Social smiling is the earliest gained social communication skill, emerging around two months of age, and is recognized as a key two-month developmental milestone by the American Academy of Pediatrics [7,8]. The described findings were noted on routine examination, without using a standardized screening tool. These early developmental red flags, in the context of congenital microcephaly, raised concern for an underlying structural brain abnormality.

Diagnostic assessment and reasoning

**Figure 3:**
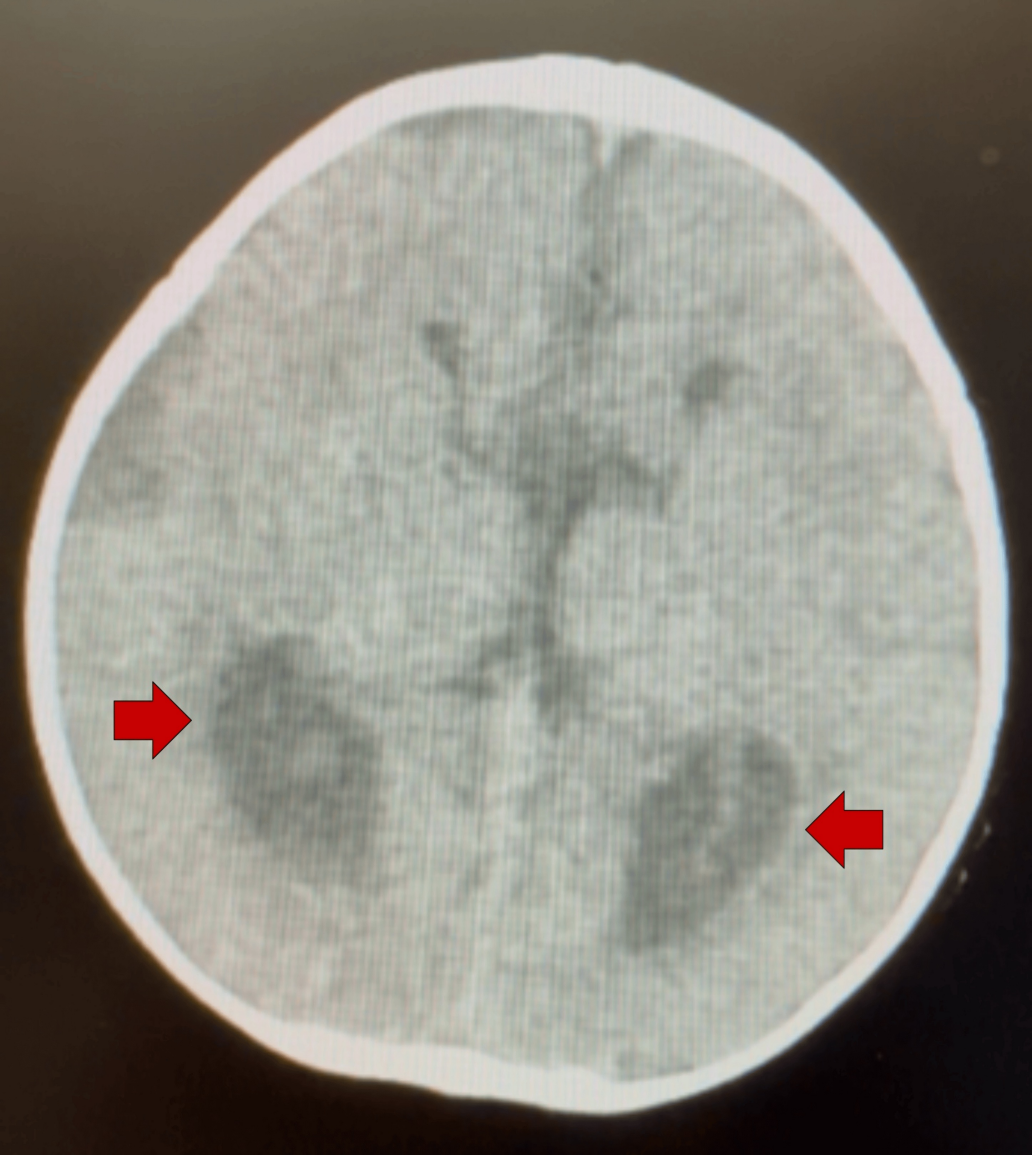
Axial CT of the brain Axial CT of the brain demonstrates widely spaced lateral ventricles with prominent dilation of the occipital horns, identifying colpocephaly (red arrows). This ventricular displacement creates a “race car” configuration characteristic of agenesis of the corpus callosum.

**Figure 4:**
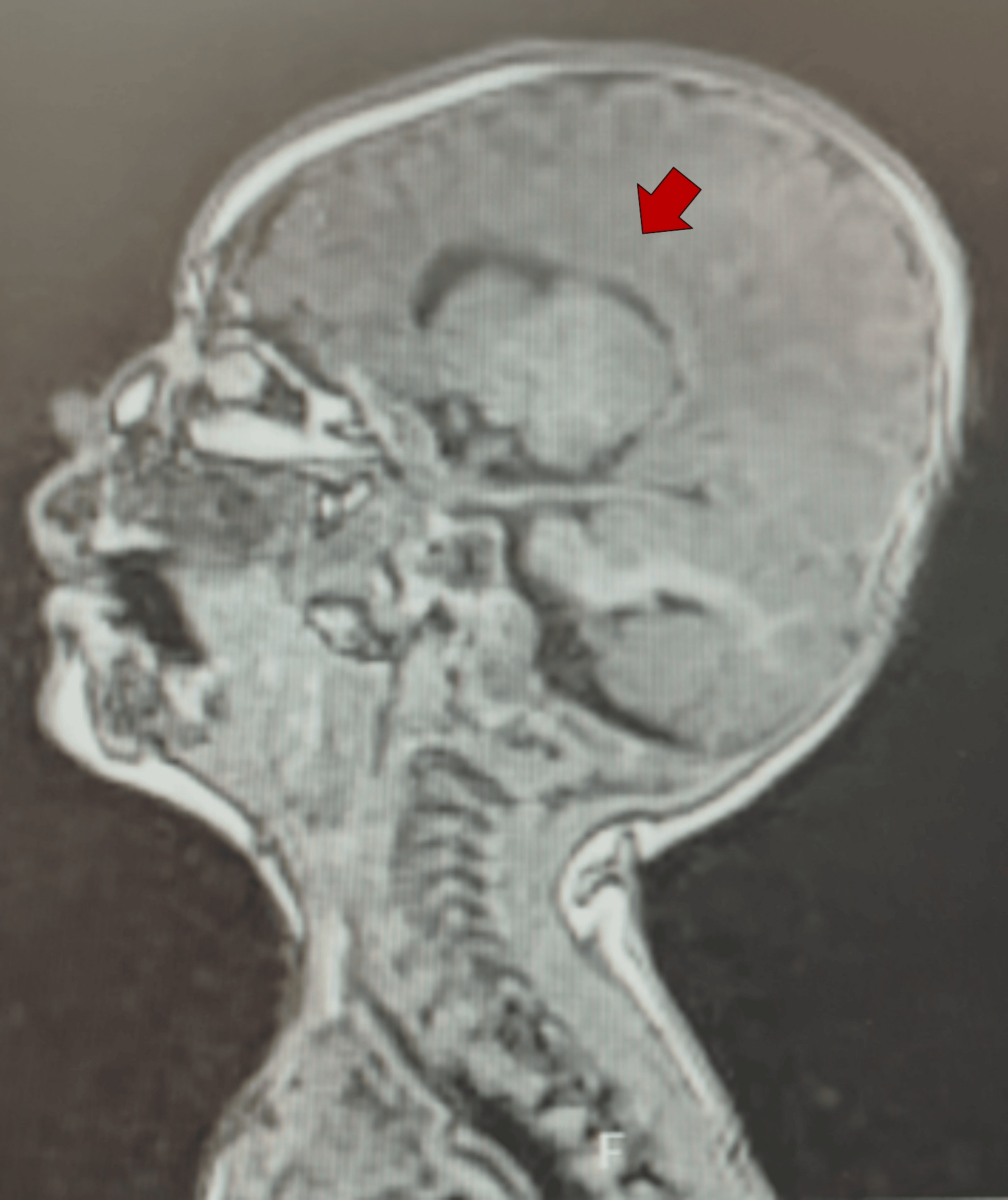
MRI scan of the brain Parasagittal MRI scan demonstrating agenesis of the corpus callosum, characterized by the absence of normal callosal fibers (red arrow). The image also reveals increased space within the posterior fossa, possibly indicative of a Blake pouch cyst.

Given the abnormal cranial findings and concern for craniosynostosis or other cranial abnormalities, the patient was referred for pediatric neurosurgical consultation. Cranial computed tomography (CT) demonstrated patent cranial sutures, which excluded primary craniosynostosis. However, the magnetic resonance imaging (MRI) impression revealed agenesis of the corpus callosum with characteristic findings, including colpocephaly (disproportionate enlargement of the occipital horns of the lateral ventricles) and parallel orientation of the lateral ventricles (Figure 3). Additionally, a possible BPC was identified in the posterior fossa (Figure 4).

The anterior cranial fossa appeared small according to the radiologist's report, consistent with reduced cerebral expansion. The neurosurgical team concluded that the cranial findings, including suture ridging and a small fontanelle, were likely related to restricted brain growth associated with the underlying brain malformations rather than premature suture closure [9].

The combination of ACC with a BPC represents complex ACC, which carries different prognostic implications than isolated ACC [3]. A BPC results from a failure of fenestration of the foramen of Magendie during embryogenesis and is part of the Dandy-Walker continuum [5]. While isolated BPC generally has favorable neurodevelopmental outcomes, with abnormal outcome rates of only 4.7% in meta-analysis, the presence of associated anomalies significantly affects prognosis [3,10]. BPC may be complicated by hydrocephalus, which requires monitoring [11].

The patient's early developmental concerns, absent social smile, and poor eye contact are particularly significant in the context of complex ACC. Research has demonstrated that children with ACC have an increased likelihood of developmental delays and autistic behaviors [4]. A recent prospective longitudinal study found that by 24 months, 29% of children with ACC were delayed in at least 1 developmental domain, with communication skills affected as early as 6 months of age [4]. Furthermore, complex ACC (with associated brain abnormalities or genetic diagnoses) is associated with significantly higher rates of motor delay, language delay, and cognitive delay compared to isolated ACC [12].

Therapeutic intervention

At the time of this report, the patient has not required surgical intervention due to the absence of true craniosynostosis. Management focuses on supportive care for feeding difficulties and initiating appropriate subspecialty evaluations. Recommended interventions include referrals to pediatric neurology for a comprehensive developmental assessment and seizure surveillance, and to pediatric ENT for suspected laryngomalacia and a feeding evaluation. A genetic evaluation incorporating chromosomal microarray analysis and potentially whole exome sequencing was also advised. Additionally, providers recommended an echocardiogram to evaluate for cardiac anomalies, given the association between posterior fossa malformations and congenital heart disease [3]. Finally, recommendations included an ophthalmologic evaluation to assess visual function and rule out associated anomalies [13], alongside an early intervention referral prioritizing communication and social development.

Follow-up and outcomes

The patient is scheduled for pediatric neurology and ENT follow-up for ongoing developmental monitoring and to assess for additional structural abnormalities. Parents report they have a scheduled appointment with a pediatric gastroenterologist, ophthalmologist, and geneticist in the next few weeks for a multidisciplinary approach to their son’s care. Genetic testing was deferred until the family could meet with a genetic counselor. No seizures have been observed to date. The family has been counseled regarding the variable prognosis associated with complex ACC and the importance of ongoing neurodevelopmental monitoring.

## Discussion

This case illustrates a diagnostic challenge in early infancy in which cranial suture ridging, slow cranial growth, and microcephaly raised concern for craniosynostosis or other cranial abnormalities. However, imaging revealed patent sutures and multiple structural brain abnormalities, highlighting the importance of distinguishing primary cranial suture pathology from secondary cranial growth restriction. Additionally, this case underscores the importance of recognizing early developmental warning signs in infants with congenital brain malformations.

Cranial development is closely linked to brain expansion. During normal development, the growing brain exerts outward pressure on the skull, allowing cranial sutures to remain open and permitting expansion of the cranial vault. When cerebral growth is impaired, cranial growth may also be restricted despite open sutures [7]. This phenomenon can lead to cranial shape abnormalities that clinically resemble craniosynostosis.

Agenesis of the corpus callosum occurs when commissural fibers fail to cross the midline during embryologic development, typically between 18 and 20 weeks of gestation [2]. The condition may occur as an isolated finding or in association with other structural brain abnormalities, genetic syndromes, or metabolic conditions [2,3]. Clinical outcomes vary widely depending on associated abnormalities. A meta-analysis found that isolated ACC is associated with normal neurodevelopmental outcome in approximately 75% of cases, while complex ACC with additional anomalies has significantly worse outcomes [14].

Early developmental concerns in ACC

The absent social smile and poor eye contact observed in this patient warrant particular attention. Social smiling is the earliest gained social communication skill, emerging around two months of age, and represents an important early milestone in social-emotional development [7,8]. Reduced eye gaze and facial engagement during caregiver interactions have been identified as early markers of neurodevelopmental concern, with persistent differences in social engagement observed by 6 months in infants later diagnosed with autism spectrum disorder [15]. Notably, socialization skills were relatively preserved in isolated ACC compared to other developmental domains, suggesting that the social deficits observed in this patient may indicate more severe involvement or the presence of additional contributing factors [4].

The presence of additional brain anomalies, complex ACC, significantly impacts the developmental trajectory in children with isolated ACC. A retrospective cohort study of 161 children with ACC found that complex ACC was associated with significantly higher rates of motor delay, language delay, cognitive delay, epilepsy, and need for assistive devices compared to isolated ACC [12]. Approximately 50% of children with ACC in this cohort had genetic variants associated with neurodevelopmental disorders, and over 50% of those with developmental delays also had identified genetic anomalies [12].

Children with ACC have also been shown to have vulnerabilities in executive function and social cognition, with particular difficulties on complex and novel tasks [16]. These neuropsychological features may contribute to the social engagement difficulties observed in some affected infants.

BPC and posterior fossa malformations 

A BPC represents the persistence of Blake's pouch, a normal embryonic structure that typically fenestrates to form the foramen of Magendie by the end of the first trimester [5]. When fenestration fails, the pouch persists as a cystic structure in the posterior fossa. BPC is distinguished from a Dandy-Walker malformation by the presence of a normal vermis and normal-sized cisterna magna [3]. The clinical spectrum of BPC ranges from asymptomatic incidental findings to symptomatic hydrocephalus requiring intervention [11]. In a study of posterior fossa malformations, isolated BPC was associated with normal neurodevelopmental outcome in over 90% of survivors without associated anomalies [17]. Although the presence of a BPC in our patient could not be confirmed, our patient did have abnormalities in the posterior fossa that resemble a BPC.

The co-occurrence of ACC with a BPC in this patient suggests a broader disruption of midline brain development during early embryogenesis. Both structures develop during overlapping gestational periods, and their combined presence may indicate a common underlying etiology. Genetic evaluation is particularly important in such cases, as chromosomal abnormalities and pathogenic copy number variants have been identified in a significant proportion of patients with posterior fossa malformations [2,18]. In one study, 31.8% of patients with BPC had chromosomal abnormalities, including aneuploidy and pathogenic CNVs [18].

Colpocephaly, the disproportionate enlargement of the occipital horns of the lateral ventricles, is a characteristic finding in ACC resulting from the underdevelopment of the posterior white matter tracts that normally cross through the corpus callosum [3]. The presence of colpocephaly in this patient is consistent with the diagnosis of ACC and does not represent hydrocephalus requiring intervention. 

Epilepsy risk and monitoring

Children with ACC have an elevated risk of epilepsy, with rates of approximately 44% in one series of children with ACC and interhemispheric cysts [19]. Epilepsy was significantly associated with cognitive impairment in this population [19]. In children with complex ACC, EEG showed specific epileptic activity in 75% of patients, though not all developed clinical seizures [19].

Clinical implications

In this patient, microcephaly and restricted cranial growth reflect reduced cerebral expansion secondary to the underlying malformations. Feeding difficulties and possible laryngomalacia may suggest broader neurologic involvement affecting brainstem function. The early developmental concerns, including an absent social smile and poor eye contact, raise concern for early global developmental delay that may be more severe than typical for isolated ACC.

Identification of complex ACC during evaluation for a cranial deformity underscores the importance of neuroimaging in infants presenting with abnormal skull morphology and growth restriction. Early recognition of the underlying neurologic etiology is essential for guiding management, including developmental monitoring, neurologic evaluation, and genetic assessment when appropriate. Distinguishing secondary cranial restriction from true craniosynostosis avoids unnecessary surgical intervention and allows for appropriate counseling regarding prognosis.

Prevention

Although the etiology of ACC remains undetermined in many cases, several environmental and infectious causes represent an important and potentially preventable subset. Awareness of these modifiable risk factors is essential for clinicians counseling families and for public health efforts aimed at reducing the incidence of congenital brain malformations.

Congenital infections with the strongest evidence for causing microcephaly and corpus callosum abnormalities include cytomegalovirus (CMV), herpes simplex virus, rubella, Toxoplasma gondii, and Zika virus [20,21]. CMV is of particular concern, as infection at any point during pregnancy can result in severe CNS sequelae, including ventriculomegaly, intracranial calcifications, and callosal dysgenesis [20]. Zika virus is intensely neurotropic, targeting neural progenitor cells and producing a severe spectrum of CNS abnormalities, including microcephaly, cortical thinning, and ACC [20,22]. Prenatal screening for TORCH (toxoplasmosis, others (syphilis, hepatitis B), rubella, cytomegalovirus, herpes simplex) infections and counseling regarding Zika virus exposure in endemic areas remain important components of comprehensive prenatal care.

Environmental teratogens associated with noninfectious causes of microcephaly include in utero exposure to alcohol, ionizing radiation, and certain medications [21]. Secondary microcephaly can arise from placental inflammation and intrauterine vascular insufficiency, resulting in brain development disruption due to hypoxic-ischemic injury and subsequent fetal malnutrition [23].

Intrauterine inflammation, whether triggered by infection or other maternal immune activation, is increasingly recognized as a contributor to microcephaly pathogenesis [23,24]. Maternal immune activation (MIA) elevates inflammatory cytokines, IL-6, IL-1β, and IL-17A, which can cross the placenta barrier, impairing fetal brain development by suppressing neural progenitor proliferation and disrupting neuronal migration [23]. Dysregulated neurogenesis results in an increased risk for fetal neurodevelopmental disorders, including schizophrenia, ADHD, or autism spectrum disorder [23]. Severe inflammatory responses have been linked to stillbirths, preterm births, and microcephaly [23,24].

Early detection of CNS anomalies through standardized prenatal ultrasound allows for timely genetic testing, prognostic counseling, and perinatal planning. While approximately one-third of CNS anomalies can be suspected in the first-trimester ultrasound, ACC is typically not identified until later in pregnancy [2]. When structural brain abnormalities are detected, chromosomal microarray analysis (CMA) is recommended, or whole exome sequencing (WES) if CMA is normal [2].

Strengths and limitations

This case report has several strengths. The patient underwent a comprehensive diagnostic evaluation, including both CT and MRI, allowing definitive exclusion of craniosynostosis and detailed characterization of the underlying brain malformations. The detailed documentation of early developmental concerns, including absent social smile and poor eye contact, provides important clinical information for prognostication and intervention planning. The clinical presentation illustrates an important diagnostic pitfall with educational value for pediatricians, neurosurgeons, and other clinicians who evaluate infants with abnormal head growth.

Limitations include those inherent to a single case report, which limits generalizability. A limitation of this case is the incomplete perinatal history, as medical records from the home birth were unavailable. This precluded review of original prenatal imaging and documentation of potential infectious or teratogenic exposures. Additionally, the suspected BPC requires further characterization to distinguish it from other posterior fossa anomalies, such as Dandy-Walker malformation, which may have different prognostic implications. Genetic testing results are pending at the time of this report, and long-term neurodevelopmental outcomes are not yet available.

Patient perspective

The patient's family expressed initial concern regarding their child’s decline in growth, especially in comparison to their older son, who had no developmental delays or growth concerns. Following the diagnosis of agenesis of the corpus callosum, the family expressed uncertainty regarding their child's developmental prognosis. They have been receptive to referrals for early intervention services and genetic counseling.

## Conclusions

This case underscores the critical diagnostic distinction between primary craniosynostosis and secondary cranial growth restriction in infants presenting with microcephaly, suture ridging, and failure to thrive. As demonstrated, underlying structural brain malformations can impair cerebral expansion, leading to secondary cranial restriction even when sutures remain patent. Furthermore, the emergence of early developmental red flags, notably the absence of a social smile and poor visual engagement, alongside congenital microcephaly, should strongly elevate clinical suspicion for central nervous system anomalies. Definitive neuroimaging is therefore essential to prevent unnecessary surgical interventions and to identify conditions like complex ACC, which are associated with varying prognoses and elevated risks for developmental delays. Ultimately, early and accurate diagnosis is vital for facilitating comprehensive multidisciplinary care, appropriate genetic evaluation, and targeted neurodevelopmental monitoring.
